# Microglia and meningeal macrophages depletion delays the onset of experimental autoimmune encephalomyelitis

**DOI:** 10.1038/s41419-023-05551-3

**Published:** 2023-01-12

**Authors:** Alejandro Montilla, Alazne Zabala, Marco Er-Lukowiak, Björn Rissiek, Tim Magnus, Noelia Rodriguez-Iglesias, Amanda Sierra, Carlos Matute, María Domercq

**Affiliations:** 1 Achucarro Basque Center for Neuroscience and Department of Neuroscience, University of the Basque Country UPV/EHU, E-48940 Leioa, Spain; 2 Centro de Investigación Biomédica en Red de Enfermedades Neurodegenerativas (CIBERNED), Leioa, Spain; 3 Department of Neurology, University Medical Center Hamburg-Eppendorf, Hamburg, Germany, 20251 Hamburg, Germany; 4 Ikerbasque Foundation, E-48009 Bilbao, Spain

**Keywords:** Multiple sclerosis, Multiple sclerosis

## Abstract

In multiple sclerosis and the experimental autoimmune encephalomyelitis (EAE) model, both resident microglia and infiltrating macrophages contribute to demyelination as well as spontaneous remyelination. Nevertheless, the specific roles of microglia versus macrophages are unknown. We investigated the influence of microglia in EAE using the colony stimulating factor 1 receptor (CSF-1R) inhibitor, PLX5622, to deplete microglial population and *Ccr2*^RFP/+^ *fms*^EGFP/+^ mice, to distinguish blood-derived macrophages from microglia. PLX5622 treatment depleted microglia and meningeal macrophages, and provoked a massive infiltration of CCR2^+^ macrophages into demyelinating lesions and spinal cord parenchyma, albeit it did not alter EAE chronic phase. In contrast, microglia and meningeal macrophages depletion reduced the expression of major histocompatibility complex II and CD80 co-stimulatory molecule in dendritic cells, macrophages and microglia. In addition, it diminished T cell reactivation and proliferation in the spinal cord parenchyma, inducing a significant delay in EAE onset. Altogether, these data point to a specific role of CNS microglia and meningeal macrophages in antigen presentation and T cell reactivation at initial stages of EAE.

## Introduction

Multiple sclerosis (MS) is a chronic inflammatory disease of the brain and spinal cord leading to demyelination and neurodegeneration. These outcomes are caused by immune cell infiltration across the blood–brain barrier, promoting detrimental effects such as inflammation, gliosis, and neuroaxonal degeneration [1]. In experimental autoimmune encephalomyelitis (EAE), an MS mouse model, myelin-specific T cells are peripherally activated in secondary organs, such as lymph nodes, and migrate towards the CNS interfaces to get reactivated by antigen-presenting cells (APCs) and clonally expand to effectively carry out their actions [2, 3]. Beside T cells, resident microglia, CNS-associated macrophages (CAMs, found in interfaces such as the perivascular space or the meninges) and infiltrating monocytes are associated to the development of the MS/EAE pathology. All these myeloid populations are known to participate in both beneficial and detrimental processes regarding the development of the pathology. Microglia and monocyte-derived macrophages are thought to contribute to neurodegeneration as their number correlates with the extent of axonal damage in MS lesions [4–7]. The response of microglia/macrophages may represent one of the initial steps in EAE pathogenesis, preceding and possibly triggering T-cell development and infiltration of blood-derived cells [8–12]. However, other studies indicate that microglia/macrophages response to pathological stimuli may be protective by providing neurotrophic and immunosuppressive factors and by promoting recovery [13–15]. This dichotomic behavior could be explained on the basis of different activation states [16, 17], as modulation of microglia/macrophage response determines the EAE outcome and potentiates recovery [14, 18]. On the other hand, it has been previously hypothesized that macrophages and microglia could play different roles in the pathology. Monocyte-derived macrophages associate with nodes of Ranvier and contribute to demyelination, whereas microglia appear to clear myelin debris [11]. Accordingly, microglia during EAE display a milder immunological or inflammatory response whereas infiltrated macrophages strongly contribute to the inflammatory response [19]. Nevertheless, the specific differences in the roles of resident microglia and infiltrating monocytes are still unknown, due to the difficulty in distinguishing these two populations [20].

Microglia and CAMs, but not peripheral monocytes, arise from early progenitors in the embryonic yolk sac, that migrate and colonize the CNS, in a process controlled by the colony-stimulating factor-1 receptor (CSF-1R) and its ligands [21, 22]. CSF-1R inhibition with PLX5622 leads to microglia depletion without significant effects on peripheral immune cells [23–25] and may serve as a strategy to unveil the role of these cells. In this study, we administered PLX5622 three weeks prior to the EAE induction and along the course of the EAE, to specifically study microglial implications at all disease stages.

## Materials and methods

### Animals

All experiments were performed according to the procedures approved by the Ethics Committee of the University of the Basque Country (UPV/EHU). Animals were handled in accordance with the European Communities Council Directive. All possible efforts were made to minimize animal suffering and the number of animals used. To generate *Ccr2*^RFP/+^*fms*^EGFP/+^ double transgenic mice, *Ccr2*^RFP/RFP^ and *fms*^EGFP/EGFP^ mice were crossed and first-generation littermates were used. Both transgenic lines are on a C57BL/6 genetic background.

### Microglial depletion

To deplete microglia in vivo, C57BL/6 and *Ccr2*^RFP/+^*fms*^EGFP/+^ mice were fed with 1200 ppm PLX5622 (Plexxikon Inc.) *ad libitum*, as previously described [23–26]. Respective control animals received standard chow instead. Mice were fed for 21 days prior to further experimental procedures to ensure maximal microglial depletion [23, 24].

### EAE induction

EAE was induced in 8- to 10-week-old female C57BL/6 and *Ccr2*^RFP/+^*fms*^EGFP/+^ mice. Mice were immunized with 200 µg of myelin oligodendrocyte glycoprotein 35–55 (MOG_35–55_; MEVGWYRPFSRVVHLYRNGK) in incomplete Freund´s adjuvant (IFA; Sigma) supplemented with 8 mg/ml *Mycobacterium tuberculosis* H37Ra (Fisher). Pertussis toxin (500 ng; Sigma) was injected intraperitoneally on the day of immunization and 2 days later. Neurological score was assessed daily and ranged from 0 to 8 as previously described [27]. Depending on the experiment, mice were euthanized at different phases of the model, which are defined as pre-onset (day post-immunization (dpi) 8-9), onset (dpi 10–14) and chronic phase (dpi 25–35).

After EAE, mice were euthanized and the different organs were dissected out and processed distinctly, depending on the subsequent analysis. For real-time quantitative PCRs (qPCRs), immunohistochemistry and blood-brain barrier analysis at EAE pre-onset or onset we used the same set of mice. We isolated and immediately flash frozen the cervical and thoracic regions of the spinal cord, the spleen and the lymph nodes, and we drop-fixed the lumbar fraction of the spinal cord in 4% paraformaldehyde (PFA). Some mice were injected with Evans Blue for blood-brain barrier analysis. Finally, flow cytometry experiments were performed on the whole spinal cord, the spleen and peripheral blood in another set of mice. The latter was extracted right after the euthanasia and collected in lithium-heparin tubes to avoid coagulation.

### Immunohistochemistry

In order to perform histological analysis on the spinal cords after EAE, tissues were fixed in 4% PFA for 3–4 h, and subsequently transferred to 15% sucrose for at least 2 days. Then, lumbar regions were frozen in 15% sucrose - 7% gelatine solution in PBS, and 12-µm coronal sections were obtained. Primary antibodies used for immunohistochemistry were: mouse anti-myelin basic protein (MBP) (1:1000; #808401 BioLegend), rabbit anti-MBP (1:200; #AB980 Millipore), rabbit anti-Iba1 (1:500; #019-19741 Wako Chemicals), mouse anti-GFAP (1:40; #MAB3402 Millipore), rabbit anti-mannose receptor (1:200; #ab64693 Abcam), rat anti-CD3 (1:50; #MCA1477 Bio-Rad), rat anti-CD45R (1:200; #557390 BD Bioscience), rat anti-Ly6G (1:100; #127601 BioLegend), mouse anti-SMI32 (1:1000; #801701 BioLegend), rabbit anti-Ki67 (1:500, #VP-RM04 Vector Laboratories), mouse anti-CD31 (1:100; #sc376764 Santa Cruz), rat anti-GFP (1:200; #GF090R Nacalai) and rabbit anti-dsRed (1:600; #632496 Takara). The anti-GFP and anti-dsRed antibodies were used to amplify the intrinsic fluorescent signals in the *Ccr2*^RFP/+^*fms*^EGFP/+^ mice. These primary antibodies were subsequently detected by incubation with appropriate Alexa Fluor 488 or 594 conjugated goat antibodies (1:250; Invitrogen). Moreover, cell nuclei were stained using Hoechst 33258 (1.5 µg/mL, #861405 Sigma-Aldrich). Images were acquired using a Leica TCS STED SP8 confocal microscope or a Zeiss LSM800 confocal microscope with the same settings for all samples within one experimental group. White matter lesions were defined and delimitated in figures on the basis of loss or damage to myelin (MBP immunostaining) and on the basis of the accumulation/infiltration of immune cells (Hoechst staining) in the same or on consecutive sections. The number of infiltrating immune cells was counted in three sections of 3–5 different mice and results were normalized to the total white matter area of each section. GFAP immunoreactivity, indicative of astrogliosis, was quantified in ROIs defined in white matter lesions, in non-lesioned white matter and in gray matter and normalized to ROI area. All the image analysis was performed with the ImageJ software (National Institutes of Health).

### Evaluation of blood–brain barrier disruption

To analyze whether the blood–brain barrier permeabilization was different in the experimental groups at the pre-onset stage EAE, mice were immunized against MOG peptide and sacrificed at dpi 8–9. Briefly, mice were injected intraperitoneally with 2% Evans Blue (EB) in saline (200 µL), and let it spread throughout the body for 1 hour prior to euthanasia. Spinal cord tissue was fixed and processed as described above, and EB staining in blood vessels was evaluated by immunohistochemistry.

### Flow cytometry

For the fluorescence-activated single cell (FACS) analysis, immune cells were isolated from spleen, spinal cord, and peripheral blood and processed differentially. Specifically, spleens were mashed through 70 µm cell strainers using a syringe piston. Alternatively, spinals cords were processed by both enzymatical and mechanical digestion and nuclear cells were isolated from the debris using a Percoll gradient. Peripheral blood was processed as described in the “EAE induction” section. During the protocol, erythrocytes were lysed using an ACK lysis buffer (155 mM NH_4_Cl, 10 mM KHCO_3_, 0.1 mM EDTA, pH 7.2).

Single-cell suspensions were incubated with TruStain FcX™ (anti-mouse CD16/32) antibodies for 15 min to block unspecific bindings, and then stained for 30 min with fluorochrome-conjugated monoclonal antibodies in buffer containing 1 mM EDTA (Sigma) and 0.1% bovine serum albumin (Sigma). Regarding the immune cell profiling, the antibodies used were the following: CD3-PE/Cy7 (#100220), CD11b-Bv510 (#101263), CD8-Bv650 (#100742), CD4-Bv785 (#100453), CD80-PE (#104708), CD11c-Bv605 (#117334), CD45-APC/Cy7 (#103116), Ly6G-AF700 (#127622), TCRgd-perCP/Cy5.5 (#118118), CD86-Bv421 (#105032), P2Y12-APC (#848006) and MHCII-FITC (#107606). All these antibodies were used in a 1:100 concentration and were acquired from BioLegend. The staining cocktails were designed to minimize the effects of spectral overlap. Moreover, a compensation matrix was calculated and applied to the obtained signals. Cells were analyzed using a BD FACSCelesta, and all the data was analyzed with FlowJo software (BD Bioscience).

Regarding the measurement of resident microglia and invading macrophages populations in spinal cord during EAE chronic phase, we used CD11b-FITC (1:200; #101206 BioLegend) and CD45-PE (1:100; #103106 BioLegend) as antibodies, identifying microglia as the CD11b^+^/CD45^low^ population, and infiltrating monocytes as the CD11b^+^/CD45^hi^ population. In addition, CD11b^-^/CD45^high^ population was identified as lymphocytes. Spinal cords were processed as described above. These analyses were performed using a BD FACSJazz cell sorter and analyzer.

### Serum cytokines quantification

In parallel to the FACS analysis, part of the serum from both control and PLX5622-treated animals’ blood was separated in order to measure different pro- and anti-inflammatory cytokines. Specifically, the levels of IFN-γ, TNF-α, IL-2, IL-6, IL-17A, and IL-22 were measured using a LEGENDplex™ Mouse Th Cytokine Panel (BioLegend), according to the manufacturer’s instructions.

### Quantitative RT-PCR

Total RNA from EAE lumbar spinal cords, spleens and lymph nodes was isolated using TRIzol (Invitrogen) according to the manufacturer’s instructions. Afterwards, 2 µg of this RNA was used to perform a retrotranscription protocol, using SuperScript III Reverse Transcriptase (200 U/μL; Invitrogen) and random hexamers as primers (Promega).

Real-time quantitative PCRs (qPCRs) were conducted in a Bio-Rad Laboratories CFX96 real-time PCR detection system, as previously described [28]. The reactions were performed using the iTaq Universal SYBR Green Supermix (#1725120 Bio-Rad), that includes SYBR Green as DNA-binding dye and iTaq DNA polymerase. The specific primers for different T cell subtypes are depicted in Table 1. These primers were designed using Primer Express Software (Applied Biosystems) at exon junctions to avoid genomic DNA amplification. The cycling conditions comprised 3 min of polymerase activation at 95 °C and 40 cycles consisting of 10 s at 95 °C and 30 s at 60 °C. The amount of cDNA was quantified using a standard curve from a pool of cDNA obtained from the different conditions of the experiment. Finally, the results were normalized using a normalization factor based on the geometric mean of housekeeping genes (Table 1) obtained for each condition using the geNorm v3.5 software [29].

**Table 1:** Sequences of primers used for qPCRs.

Target gene	Forward sequence (5′->3′)	Reverse sequence (5′->3′)
*Foxp3*	ACCACACTTCATGCATCAGCTC	GGCTGGGTTGTCCAGTGGAC
*Ifng*	TAACTATTTTAACTCAAGTGGCATAGATGTG	GCCAGTTCCTCCAGATATCCAAG
*Ror*	ACTGAAAGCAGGAGCAATGGAAG	TTCAAAAAAGACTGTGTGGTTGTTG
**Housekeeping gene**	**Forward** **sequence** **(5′->3′)**	**Reverse** **sequence** **(5′->3′)**
*Actin*	CCAGCCTTCCTTCTTGGGTATG	AACGCAGCTCAGTAACAGTCCG
*B2m*	ACTGACCGGCCTGTATGCTA	ATGTTCGGCTTCCCATTCTCC

### Statistical analysis

Data are presented as mean ± SEM with sample size and number of repeats indicated in the figure legends. All the statistical analyses were performed with GraphPad Prism 8.0 (GraphPad Software), including a prior test for homoscedasticity and normality. Comparisons between two groups were analyzed using unpaired Student’s two tailed *t* test, except in MOG-EAE experiments where statistical significance in neurological score was determined by Mann-Whitney *U* test. Comparisons among multiple groups were analyzed by one-way analysis of variance (ANOVA) followed by Bonferroni’s multiple comparison tests. Statistical significance was considered when *p* < 0.05.

## Results

### PLX5622 treatment causes a delay in the EAE onset and massive infiltration of macrophages

**Fig. 1:**
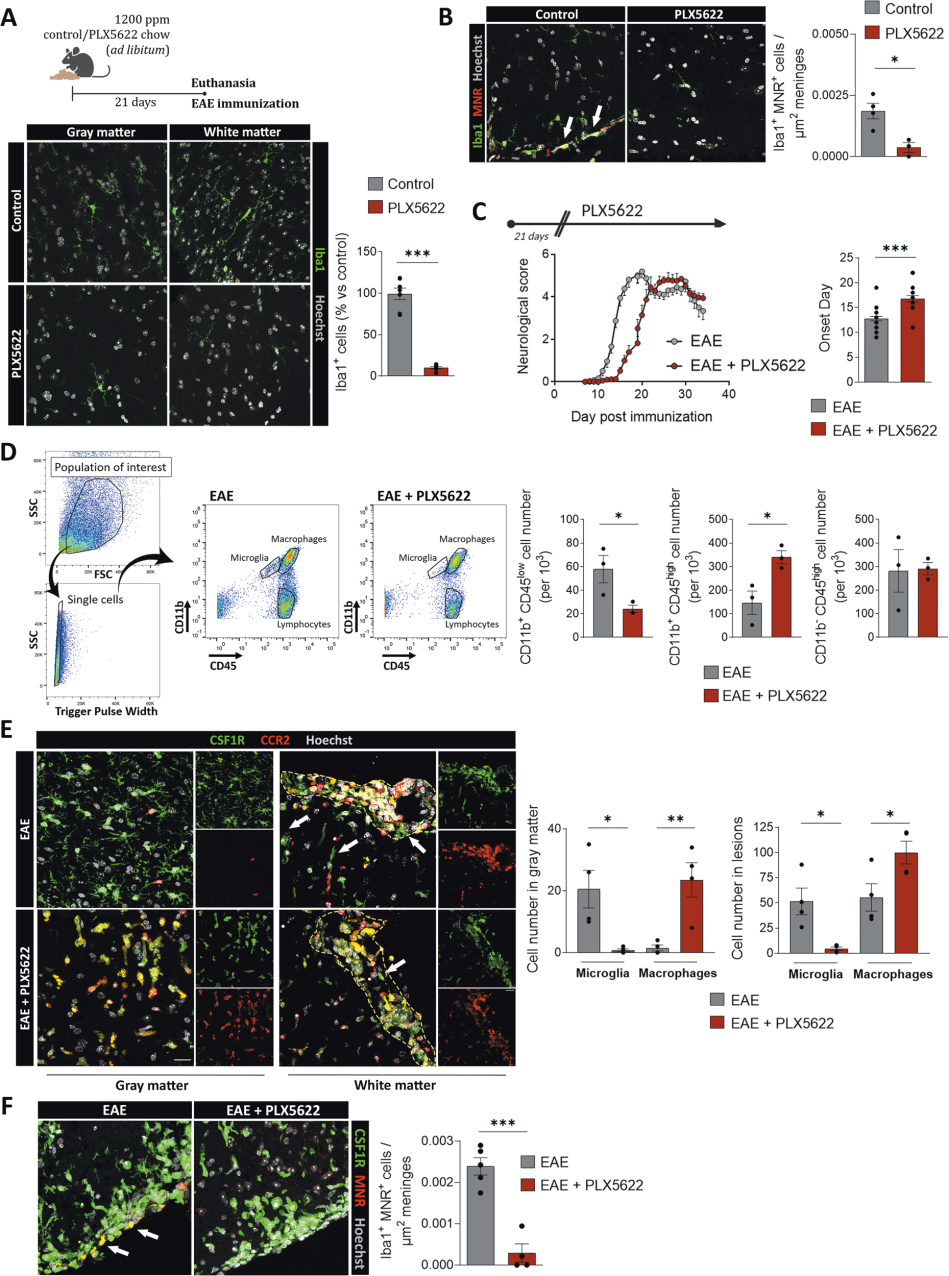
PLX5622 microglial depletion provokes a delay in the onset of EAE and massive infiltration of macrophages. **A** (*Up*) Scheme showing the paradigm of microglial depletion with PLX5622. (*Down*) Representative images showing microglial (Iba1^+^ cells) depletion in both white and gray matter of healthy spinal cord. Histogram shows the loss of microglia after PLX5622 treatment, in comparison to control mice (*n* = 5). Scale bar = 25 µm. **B** Representative images of Iba1^+^ MNR^+^ meningeal macrophages in the spinal cord of control mice and mice after complete PLX5622 treatment. Histogram shows the loss of meningeal macrophages after PLX5622 treatment, in comparison to control mice (*n* = 4). Scale bar = 25 µm*.* **C** (*Left*) Neurological score of control and PLX5622-treated mice, (*n* = 20–25 mice from three independent EAE experiments). (*Right*) Histograms showing the onset day of clinical signs of every mouse (other clinical parameters are shown in Supplementary Fig. 1) **D** Plots depicting the strategy to distinguish resident microglia (CD11b^+^/CD45^low^), invading macrophages (CD11b^+^/CD45^hi^) and lymphocytes (CD11b^-^/CD45^hi^) in EAE spinal cords from control and PLX5622-treated mice. Histograms show the quantification of all the populations, in relation to the total number of analyzed cells in the samples (*n* = 3 mice per group) **E** Representative images of *fms*^+^ microglia (green, arrows) and *Ccr2*^+^ macrophages (red) in the gray and white matter of control and PLX5622-treated *Ccr2*^*RFP/+*^*fms*^*EGFP/+*^ mice at EAE chronic phase (dpi 30–35). Dashed line delineates white matter lesions. Histograms at the right show the quantification of microglia (fms^+^ CCR2^−^ cells) and macrophages (CCR2^+^ cells) in each region (*n* = 4). Scale bar = 20 µm. **F** Representative images of *fms*^*+*^ MNR^+^ meningeal macrophages (arrows) in the spinal cord of control and PLX5622-treated EAE mice. Scale bar = 25 µm. Histogram shows the number of meningeal macrophages in relation to the area of the meninges analyzed (*n* = 5). Data are presented as means ± SEM. *p < 0.05, ***p < 0.001.

Following EAE induction, CNS-resident microglia and invading macrophages contribute to disease pathogenesis, influencing both progression and recovery of the disease [30]. To address the role of microglial cells at all EAE stages, we used the CSF-1R-specific inhibitor PLX5622 (administered at 1200 ppm in chow) to deplete microglia. We eliminated 90% of microglia throughout the lumbar spinal cord after 3 weeks of treatment, in a similar proportion to that achieved in other studies [24, 31] (Fig. 1A). We further characterized whether PLX5622 treatment affected CNS-associated macrophages, which could be distinguished from microglia by the expression of mannose receptor C-type1 (MRC1, also known as CD206) [32]. In control mice, PLX5622 treatment also reduced the number of Iba1^+^ MNR^+^ cells at the meninges (Fig. 1B), demonstrating that PLX5622 also depleted meningeal macrophages.

Induction of EAE after treatment with PLX5622 for 3 weeks delayed the onset of symptoms but not the subsequent disease course (Fig. 1C). We did not observe differences in EAE induction, severity or in the recovery capacity during EAE chronic phase, 30–35 days post-immunization (dpi 30–35) (see supplementary Fig. 1). Then, we assessed the populations of microglia (CD11b^+^ CD45^low^) and invading macrophages (CD11b^+^ CD45^hi^) at the end of the disease by FACS, and we observed that the ablation of microglia was maintained throughout the EAE (Fig. 1D). No changes were detected in lymphocytes (CD11b^-^ CD45^hi^) (Fig. 1D).

To understand the dynamics of CNS microglia, meningeal macrophages, and monocyte-derived macrophages, we induced EAE in control and PLX5622-treated *Ccr2*^RFP/+^*fms*^GFP/+^. These mice allowed us to distinguish resident microglia and CAMs (*Ccr*2^-^*fms*^+^) vs infiltrating macrophages (*Ccr*2^+^ *fms*^-^) on spinal cord sections. Of note, infiltrated macrophages (*Ccr*2^+^) started to express *fms-EGFP*, so we also considered *Ccr*2^+^*fms*^+^ cells as infiltrating macrophages. Indeed, the CNS environment induces a microglial-like phenotype in myeloid populations [33]. PLX5622 chronic treatment in these mice (as described before) induced a massive reduction of *Ccr*2^-^ *fms*^+^ microglia cells as well as *Ccr*2^-^ *fms*^+^ CD206^+^ meningeal macrophages at EAE chronic phase (35 days post-immunization: Fig. 1C, E, F). In contrast, we observed an increase in peripheral macrophages in the white matter demyelinated lesions in PLX5622-treated mice (Fig. 1E). In addition, macrophages penetrated into the non-damaged white matter and even into the gray matter in PLX5622-treated mice (Fig. 1E). These results demonstrate that resident microglia and meningeal macrophages limit the massive entry of macrophages and their dispersion through the CNS parenchyma in response to pathological conditions. Interestingly, despite the massive infiltration of monocyte-derived cells, microglia and meningeal macrophage depletion did not trigger an exacerbated progression of EAE, nor did it cause a failure in the recovery, as described earlier [9]. Together, these results indicate that microglial and meningeal macrophage ablation provokes a compensatory mechanism based on a robust infiltration of peripheral macrophages along with a delayed disease onset.

### Microglia and meningeal macrophages depletion does not alter EAE chronic pathophysiology

Microglial function during EAE is commonly associated with its capacity to phagocytose myelin debris, promoting recovery and regeneration in the chronic phase of the disease. We further assessed whether depletion of microglia affected demyelinated lesions at this stage using immunohistochemistry. The extent of the lesioned area was determined by the accumulation of infiltrating cells, and by the loss or damage of myelin (characterized by high MBP immunoreactivity).

**Fig. 2:**
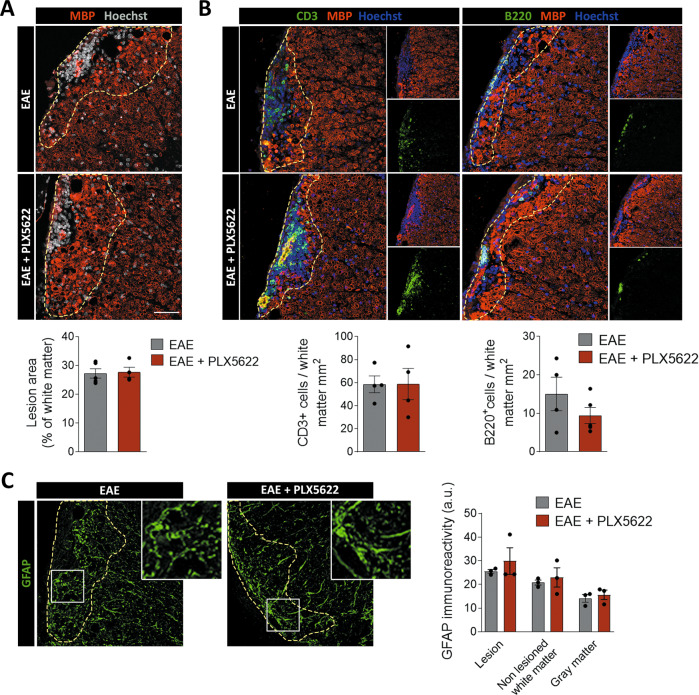
PLX5622 microglial depletion does not alter EAE chronic phase. **A** Representative images of EAE lesions in the lumbar spinal cord of control and PLX5622-treated mice (dpi 30–35). The histogram shows the percentage of lesioned white matter versus total white matter (*n* = 5). Scale bar = 50 µm. **B** Representative images showing the accumulation of CD3^+^ T cells and B220^+^ B cells in EAE lesions in control and PLX5622-treated at EAE chronic phase (dpi 30–35). Scale bar = 25 µm. Histograms show the number of T cells and B cells normalized to total white matter (*n* = 4). **C** Representative images of GFAP astrocyte immunoreactivity, indicative of astrogliosis, in lesioned and non-lesioned white matter of control and PLX5622-treated mice at EAE chronic phase (dpi 30–35; *n* = 3). Dashed lines delineate white matter lesions in all images. Insets show higher magnification of the indicated boxes. Data are presented as means ± SEM.

We observed no differences in the white matter area affected by EAE between control and PLX5622-treated mice (Fig. 2A). Demyelination in EAE is mediated by an immune response, mainly based on T cell activity but also on B cells infiltration [34]. Because of that, we analyzed the presence of both populations in the lesions and found no differences in meningeal and parenchymal infiltrating accumulation of both cell types (Fig. 2B). Lastly, we observed no alteration in astrogliosis neither in the lesions nor in the white matter surrounding the lesions or the gray matter (Fig. 2B).

In sum, these findings show that the course of the chronic phase of EAE was not altered in mice with depleted microglia. As we detected similar partial remission of the symptoms in both experimental groups, this suggests that microglia are not necessary for an EAE better outcome or a more efficient remyelinating process during the disease.

### Peripheral immune priming is not altered in EAE after microglial and meningeal depletion with PLX5622

As previously described, EAE initial stages primarily imply T cell activation in peripheral lymphoid organs, such as the spleen or lymph nodes, against myelin-specific peptides [2]. Since microglial ablation delayed EAE onset, we hypothesized that microglia could modulate immune priming or immune infiltration. In order to corroborate these hypotheses, we first assessed whether microglia could influence peripheral early responses right after immunization, and prior to the appearance of first motor deficits.

**Fig. 3:**
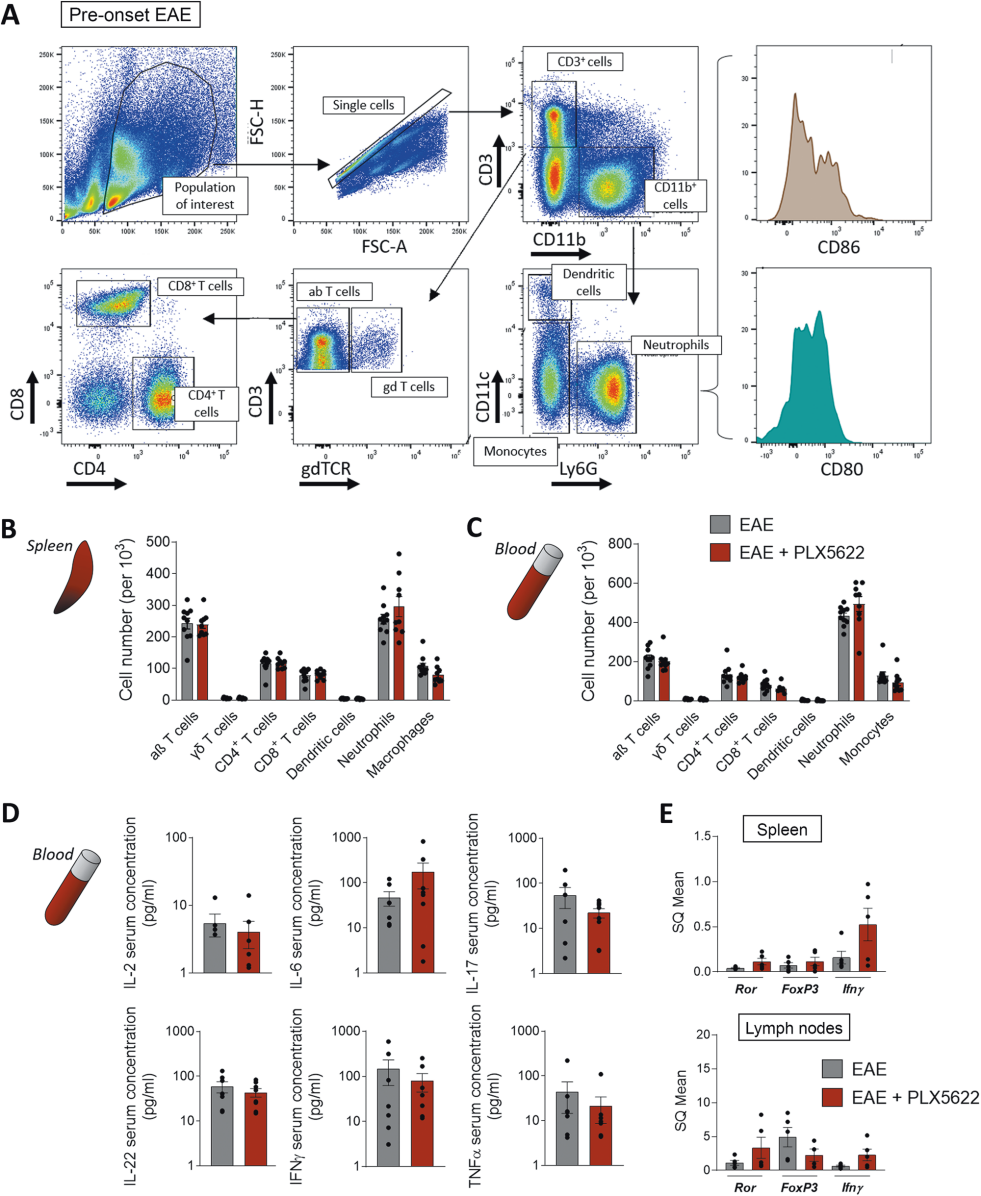
PLX5622 does not alter peripheral immune priming after immunization. **A** Flow cytometry gating strategy for analysis of immune populations in the spleen and peripheral blood of mice at EAE pre-onset (dpi 8–9). Quantification of αβ T cells (CD3^+^ γδTCR^-^), γδ T cells (CD3^+^ γδTCR^+^), CD4 T cells (CD3^+^ CD4^+^) CD8 T cells (CD3^+^ CD8^+^), dendritic cells (CD11b^+^ CD11c^+^), neutrophils (CD11b^+^ Ly6G+), and macrophages/monocytes (CD11b^+^ CD11c^-^ Ly6G^-^) populations in spleen (**B**) and blood (**C**), in relation to the total cells analyzed at this stage (*n* = 10). **D** Concentration of cytokines in blood serum from control and PLX5622-treated mice at EAE pre-onset stage (*n* = 8). **E** Relative mRNA expression of *Ror*, *Foxp3* and *Ifnγ* in spleen and lymph nodes of both mice groups, at EAE pre-onset stage (dpi 9; *n* = 5). Data are presented as means ± SEM.

We immunized mice and euthanized them at the pre-onset phase of EAE (dpi 8–9; no clinical signs). At this timepoint, we performed flow cytometry analysis of the immune cell populations in spleen and peripheral blood (gating as specified in Fig. 3A). Microglial depletion by inhibition of CSF-1R did not alter the proportion of CD4^+^ T cells, CD8^+^ T cells, γδ T cells, neutrophils or macrophages/monocytes neither in the spleen nor in the blood (Fig. 3B, C).

Taking advantage of the blood sampling, we analyzed the concentration of pro- and anti-inflammatory cytokines in the serum of both animal groups, using a bead-based immunoassay. Specifically, we measured the levels of innate and adaptive immune cytokines such as IL-2, IL-6, IL-17, IL-22, IFNγ, and TNFα, all associated with EAE pathology [35]. PLX5622 treatment did not lead to any difference in the cytokine profiles in the serum at EAE pre-onset (Fig. 3D). Moreover, as EAE model is based predominantly in a CD4^+^ T cell response, we assessed their phenotypes by qPCR analysis in spleen and lymph nodes, both tissues where T cells are early activated. We measured the levels of mRNA expression for forkhead box protein P3 (*FoxP*3), retinoic acid-related orphan receptor (*Ror*), transcription factors specifying Treg and Th17 activity respectively [36, 37], as well as *Ifn*γ, signature cytokine for Th1 cells [38]. We did not find differences in the expression of any of these markers between control and PLX5622-treated mice at EAE pre-onset (Fig. 3E).

All these results suggest that microglial and CAMs ablation does not provoke significant alterations in the primary, peripheral immune priming after EAE induction. Thus, the delay in the onset of the symptoms might be associated with an effect of PLX5622 within the CNS environment.

### Spinal cord early EAE damage is not altered after microglial and meningeal depletion

As the peripheral response was not altered in mice treated with the CSF-1R inhibitor, we next analyzed the spinal cord of both control and PLX5622-treated mice at the pre-onset stage of EAE, in order to look for early signs of alteration that would lead to symptoms delay.

**Fig. 4:**
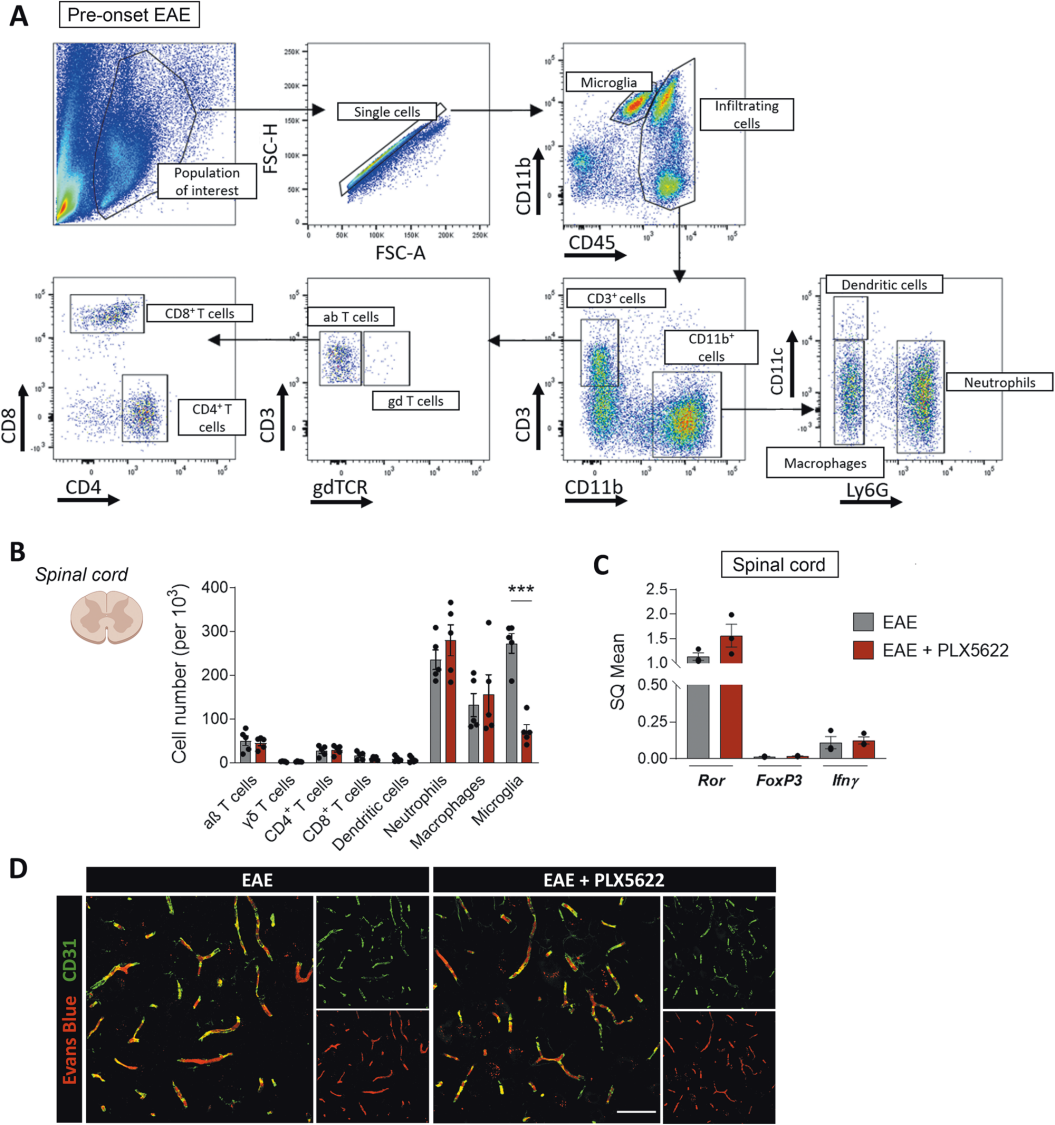
PLX5622 does not alter early, CNS-related events at the pre-onset EAE. **A** Flow cytometry gating strategy for analysis of immune populations from the spinal cord of mice at EAE pre-onset (dpi 8–9). **B** Quantification of the same populations as in the previous figure plus microglia (CD11b^+^ CD45^low^) in spinal cord, in relation to the total number of analyzed cells, at this stage (*n* = 5). **C** Relative mRNA expression of *Ror*, *Foxp3* and *Ifnγ* in the spinal cord of both mice groups, at EAE pre-onset stage (*n* = 3). **D** Representative images showing Evans Blue staining restricted to CD31^+^ blood vessels and lack of BBB disruption in the spinal cord of both control and PLX5622-treated mice. Scale bar = 50 µm. Data are presented as means ± SEM. ***p < 0.001.

We assessed immune cell infiltration into the spinal cord by flow cytometry profiling at EAE pre-onset (gating strategy specified in Fig. 4A). Aside from the expected significant reduction in the number of microglial cells in the PLX5622-treated animals, we did not identify any other differences in immune populations (Fig. 4B). Moreover, analysis of first-arriving CD4^+^ T cells’ profiles in spinal cord by qPCR did not show any differences between both experimental groups (dpi 9; Fig. 4C). Since alterations in BBB permeability are a key initiating factor promoting the infiltration of myeloid and lymphoid cells to the CNS parenchyma in MS and EAE [39], we subsequently analyzed the state of BBB integrity by histological analysis of Evans Blue in spinal cord vascularity. No differences were observed in the extravasation of Evans Blue between control and PLX5622-treated mice at EAE pre-onset (dpi 9), with all the staining remaining inside the CD31^+^ blood vessels in both groups (Fig. 4D).

These results highlight that CSF-1R inhibition does not significantly alter immune priming on the periphery at the pre-onset stage of the disease. Thus, delayed symptomatology is probably linked to a later alteration, most likely affecting events occurring within the CNS.

### Microglia and/or meningeal macrophages are key in the immune response in the CNS parenchyma

The efficient activation of T lymphocytes after arriving to the CNS parenchyma is a critical requirement for the induction of EAE pathology. Because of this, and given the delay in the appearance of motor alterations in microglial-depleted mice, we next analyzed whether microglia ablation altered the immune response at EAE onset in the CNS parenchyma. We induced EAE in control and PLX5622-treated mice and euthanized them at EAE onset, dpi 13–14.

**Fig. 5:**
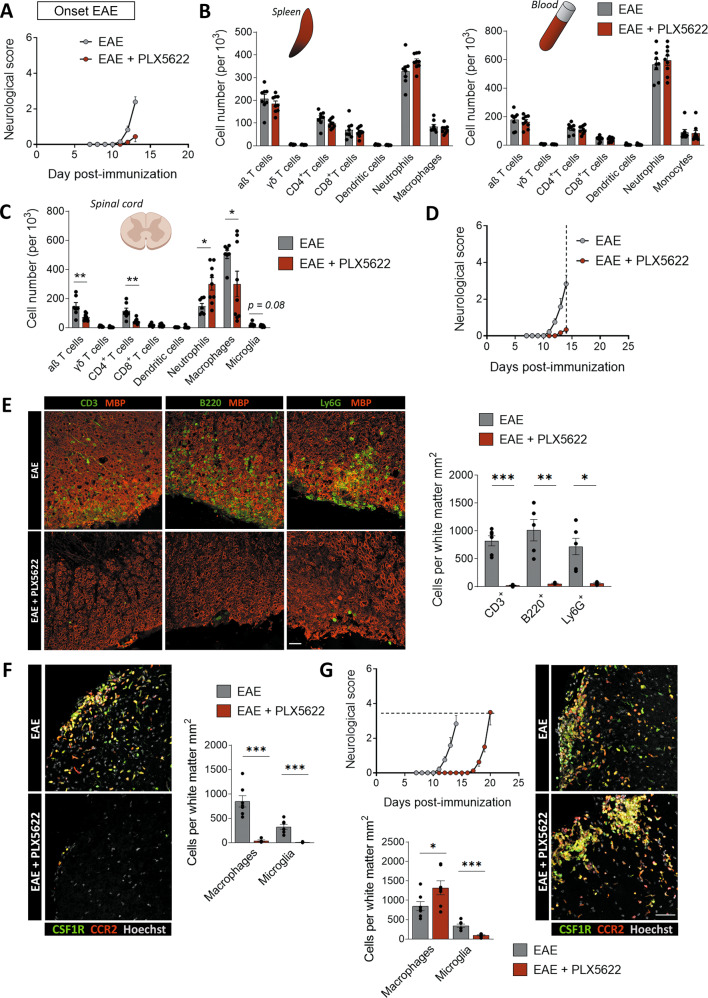
PLX5622 provokes a delay in CNS infiltration of immune cells. **A** Histogram showing the neurological score in control and PLX5622-treated mice (used for FACS analysis). **B** Quantification of immune populations in spleen and peripheral blood, in relation to the total number of analyzed cells at this timepoint (*n* = 10). Gating strategy is specified in Fig. 3A. **C** Quantification of immune populations in spinal cord, in relation to the total number of analyzed cells, at EAE onset (dpi 13–14; *n* = 10). Gating strategy is specified in Fig. 4A. **D** Histogram showing the neurological score in control and PLX5622-treated mice at dpi 14 (used for immunohistochemistry and qPCR). **E** (*Left*) Representative images of CD3^+^ T cells, B220^+^ B cells and Ly6G^+^ neutrophils infiltration into spinal cord parenchyma. (*Right*) Histogram shows the number of cells per total white matter area in control and PLX5622-treated mice (*n* = 5). Scale bar = 30 µm. Representative images and histogram showing the quantification of *fms*^+^ microglia (fms^+^ *Ccr2*^-^ cells; green) and *Ccr2*^+^ macrophages (red) in control and PLX5622-treated Ccr2^RFP/+^ fms^EGFP/+^ mice at EAE onset (dpi 14; **F**) and at a timepoint coincident with the beginning of symptoms in both groups (dpi 14 for control mice and dpi 20 for PLX5622-treated mice: **G**) (*n* = 7). Scale bar = 50 µm. Data are presented as means ± SEM. *p < 0.05, **p < 0.005, ***p < 0.001.

At this timepoint, we observed a consistent difference in the degree of pathology development (Fig. 5A). Indeed, control mice were starting to show the first motor deficiencies while PLX5622-treated mice did not. We performed flow cytometry analysis of the immune cell populations in peripheral tissues (spleen and blood); the gating strategy was carried out as previously described (Fig. 3A). These flow cytometry experiments showed no alteration in the immune cell populations in spleen and blood after PLX5622 treatment (Fig. 5B). However, in the spinal cord we detected an increased proportion of neutrophils as well as a lesser percentage of macrophages and CD4^+^ T cells in PLX5622-treated animals (Fig. 5C; gating strategy described in Fig. 4A). The larger proportion of neutrophils could be secondary to microglial depletion as microglia engulf neutrophils at the periphery of the ischemic lesion to limit its expansion in brain parenchyma [40].

We further checked immune cell infiltration by immunohistochemistry at EAE onset (dpi 14) in control and PLX5622-treated *Ccr2*^RFP/+^*fms*^GFP/+^ (see neurological score in Fig. 5D). Although the proportions of the major immune populations were not affected in microglia and meningeal macrophages depleted mice (Fig. 5C), the total number of immune cells infiltrated into the CNS parenchyma was massively reduced in PLX5622-treated mice, as revealed by immunohistochemistry (Fig. 5E). Indeed, control mice presented high levels of infiltration of T cells, B cells and neutrophils, as assessed by immunostaining of CD3, B220 and Ly6G respectively, in correlation with the neurological score (Fig. 5E) as well as macrophages (Fig. 5F). B220^+^ B cells were not restricted to the meninges but infiltrated into the white matter parenchyma, as described also before [41, 42]. Alternatively, PLX5622-treated mice showed very limited infiltration of these cells, and mostly restricted to the meninges and blood vessels (Fig. 5E, F). However, macrophage infiltration was observed at delayed times after EAE immunization (dpi 20) in PLX5622-treated mice, a timepoint coincident with the beginning of the neurological symptoms (Fig. 5G). At this stage, macrophage infiltration in white matter lesions and around the lesions is higher in PLX5622-treated mice (Fig. 5G).

These results suggest that microglia and meningeal depletion provokes a delay in the accumulation of immune cells in the CNS parenchyma, and this effect is directly linked to the adjournment of the motor deficits.

### Microglia and meningeal macrophages elimination reduced the expression of antigen-presenting proteins and T cell reactivation in EAE onset

**Fig. 6:**
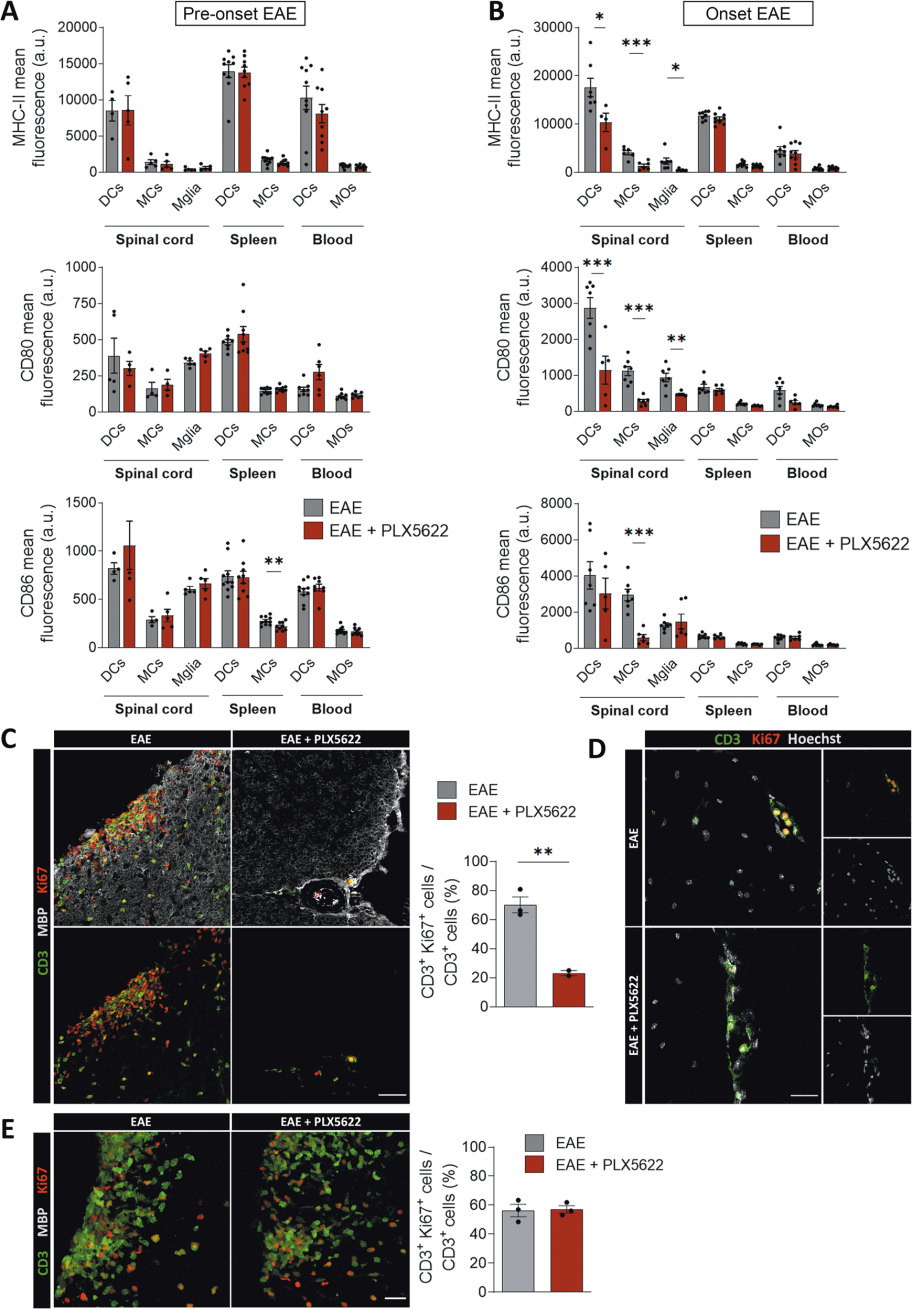
Microglial depletion with PLX5622 alters antigen presentation and T cell reactivation in the spinal cord. Histograms showing MHC-II, CD80 and CD86 fluorescence intensity in different APCs (dendritic cells (DCs), macrophages/monocytes (MCs/MOs) and microglia (Mglia)) and in diverse tissues, as analyzed by flow cytometry at the pre-onset stage of EAE (**A**; dpi 8–9) (*n* = 5 spinal cord samples, *n* = 10 spleen and blood samples), and at the onset stage of EAE (**B**; dpi 13–14) (*n* = 10). **C** Representative images showing CD3^+^ lymphocytes and Ki67 proliferative-associated expression in spinal cord, at EAE onset (dpi 14) in control and PLX5622-treated mice. Histogram show the proportion of Ki67^+^ CD3^+^ T cells in relation to the total number of CD3^+^ T cells (*n* = 3). Scale bar = 25 µm. **D** Representative images showing the early Ki67^+^ CD3^+^ proliferating lymphocytes in the spinal cord at EAE onset (dpi 10, **D**). Scale bar = 30 µm. **E** Representative images and quantification of CD3^+^ lymphocytes proliferation at a timepoint coincident with the beginning of symptoms in both groups (dpi 14 for control mice and dpi 20 for PLX5622-treated mice (*n* = 3). Scale bar = 25 µm. Data are presented as means ± SEM. *p < 0.05, **p < 0.005, ***p < 0.001.

The accumulation of T cells in the CNS tissue during neuroinflammation is commonly preceded by a reactivation step carried out by APCs. As we observed an alteration in this accumulation, we assessed the antigen presentation process both at the pre-onset and onset stages of EAE by FACS analysis. Specifically, we measured the expression of the major histocompatibility complex II (MHC-II), which plays a key role in antigen presentation and CD4 + T cell activation in EAE, and the B7 co-stimulatory molecules (CD80 and CD86), which participate along with MHC-II in this mechanism in diverse APCs [43]. At the pre-onset phase of EAE (dpi 8-9), we hardly found any significant differences regarding MHC-II, CD80 and CD86 intensity in the cell types analyzed (microglia, DCs and macrophages) in PLX5622-treated mice (Fig. 6A), except for a decrease in CD86 intensity in spleen macrophages. However, at EAE onset (dpi 13-14) we detected that the expression of MHC-II and the co-stimulatory molecules CD80 and CD86 was altered specifically in the spinal cord, but not in spleen or blood cells. The expression of MHC-II and CD80 was significantly reduced in all APCs in PLX5622-treated mice, including microglia (Fig. 6B). In addition, CD86 intensity was also significantly reduced in macrophages of PLX5622-treated mice (Fig. 6B). These findings suggest a role of microglia and meningeal macrophages in modulating antigen presentation, not only as an intrinsic mechanism, but also affecting other APC cells and their function.

As these observations suggest that antigen presentation was altered in spinal cord after microglial and meningeal macrophages depletion, and this process is needed for T clonal expansion in CNS parenchyma, we assessed whether PLX5622 affected T cell proliferation rate using antibodies to Ki67, a marker of proliferation. Control EAE mice showed a massive proliferation of immune cells inside the lesions, whereas proliferation was limited in PLX5622-treated mice at EAE onset (dpi 14; Fig. 6C; neurological score as in Fig. 5D). In particular, the number of Ki67^+^ CD3^+^ T cells was significantly reduced, suggesting that T cell proliferation and therefore their clonal expansion was delayed in mice depleted from microglia and meningeal macrophages (Fig. 6C). Of note, this effect was also observed in the first T cells arriving to the CNS after peripheral priming (Fig. 6D). However, proliferation of T cells in PLX5622-treated mice was recovered when neurological symptoms started in these mice (dpi 20; Fig. 6E; neurological score as in Fig. 5G).

Taken together, the results described in this study show that microglia and meningeal macrophages limit the dispersion of CNS-infiltrated macrophages into the CNS parenchyma. In their absence, macrophages are able to colonize the CNS parenchyma and contribute to both EAE development as well as the recovery/remyelinating mechanisms occurring in the chronic phase. On the other hand, microglia and meningeal macrophages are important to EAE development as their deletion delays immune cell activation responsible for the onset of the disease, by altering the antigen presentation in the brain.

## Discussion

Microglia participate in neurodegenerative pathologies development. Thus, the analysis of their specific role in these processes has lately emerged as an important focus of research, seeking for therapeutic approaches for diseases like MS [44]. In this study, we used the CSF-1R antagonist, PLX5622, in order to assess the effect of microglia depletion in EAE development. However, we cannot exclude that the effect observed in EAE development after PLX5622 treatment could be partly due to the elimination of meningeal macrophages. We demonstrated that microglia and meningeal macrophages interfere with peripheral macrophage dynamics by controlling their entry and migration. The massive infiltration of macrophages in the absence of microglia and meningeal macrophages does not significantly affect neurological damage at EAE peak or recovery in EAE chronic phase. However, microglia and meningeal macrophages ablation delays EAE onset demonstrating a specific role of these cells in antigen presentation and T cell proliferation at early stages of EAE.

PLX5622 has largely been assumed to be microglia-specific but more recent studies described alterations in myeloid and lymphoid populations in peripheral tissues [45, 46]. In our study, we did not find any alteration in the immune populations in spleen and blood nor in the expression of antigen-presenting proteins after PLX5622 treatment, a fact suggesting that PLX5622 did not affect directly immune cells, including peripheral monocytes. While tissue macrophages and circulating monocytes express CSF-1R, their survival is not only depending on CSF1/CSF1R signaling but relies also on CCL2/CCR2 signaling [47] which is not present in microglia. In contrast to infiltrating and peripheral macrophages, we found that CAMs, in particular meningeal macrophages, were also depleted after PLX5622 treatment. In accordance, perivascular macrophages survival is also dependent on CSF1/CSF1R signaling, as PLX5622 treatment induced a 60% reduction in their number [48].

We showed that microglial and meningeal macrophages depletion provoke a massive infiltration of CCR2^+^ peripheral macrophages during EAE progression in comparison to control mice, which could constitute a compensatory mechanism. Importantly, infiltrating macrophages in PLX5622-treated mice colonize CNS parenchyma including both white and gray matter of the spinal cord, and their location is not limited to demyelinated lesions as in control mice. These data suggest that microglia control the dispersion of macrophages throughout the CNS parenchyma. In accordance, microglia surround and confine infiltrating macrophages into the CNS parenchyma, limiting macrophage dispersion after LPC-induced demyelination [49]. In addition, we detected a higher accumulation of macrophages in demyelinated lesions after PLX5622 treatment, suggesting that microglia and meningeal macrophages also control the infiltration, proliferation, or survival of macrophages into the CNS parenchyma. In accordance, previous data using parabiosis in the EAE model demonstrated that peripheral macrophage infiltration is preceded by microglia cell death [9]. Thus, microglia could potentially help to limit peripheral CNS inflammation and to maintain the “CNS immune-privileged” status.

PLX5622 treatment induced a consistent delay in the appearance of the first EAE symptoms. This suggests that microglia and meningeal macrophages play a role in the effector stage of the disease model. Indeed, inhibition of chemokine receptor-dependent recruitment of monocytes to the CNS blocked EAE progression, not EAE onset, suggesting that microglia is essential for EAE onset whereas macrophages contribute to EAE progression [9]. Specifically, we have seen that microglia and meningeal macrophages ablation caused an alteration in antigen presentation to the first T cells arriving to the CNS, both in dendritic cells and infiltrated macrophages. Frequently, DCs have been identified as the main APCs during EAE [20, 50, 51]. Interestingly, we showed that microglial and meningeal macrophages ablation reduced the expression of the major histocompatibility complex- II, critical for the antigen-immune response, as well as the co-stimulatory molecules (CD80 and CD86) in all antigen-presenting cells, including DC cells, in the spinal cord but not periphery, excluding the possibility of a direct effect of PLX5622 treatment. This suggests microglia and/or meningeal macrophages early activation after EAE induction potentially promotes antigen presentation capacity in other cell types. This result is in accordance with those obtained in a virus model, in which PLX5622 also alters T cell local reactivation in CNS decreasing B7 co-stimulatory signals in CD11c^+^ cells [52]. Altogether, these data highlight the relevance of microglia and meningeal macrophages in orchestrating the CNS immune response.

Numerous studies determine that microglia activation occurs during the onset and peak of EAE and its activation is necessary to EAE development. Microglia control T cell encephalogenicity through the release of IL23, specifically the P40 subunit and its deletion in microglia cells suppressed EAE by shifting T cell response towards a Th2 rather than Th1 [53]. Another important signal for microglia activation is the TGFβ-activated kinase (TAK1). Thus, microglia selective ablation of TAK1 blocks its activation, the release of pro-inflammatory mediators such as IL1β and CCL2, and the immune cell infiltration, suppressing completely EAE [10]. This data is not opposite to ours as in this case microglia is still present in the brain parenchyma, a fact that could limit macrophage infiltration, as observed in our study. Overall, our data, in accordance with previous studies, indicate that microglia and its persistent and overt activation has a detrimental role in CNS autoimmunity onset, and preventing or suppressing this process may be therapeutic.

Targeting CSF-1R has also been tried in different EAE models and at different time windows leading to diverse outcomes. Inhibiting CSF-1R with PLX5622 after EAE onset attenuated EAE pathology and promoted recovery [54]. However, blocking CSF-1R with PLX3397 at later stages in EAE exacerbated neuroinflammation and neurological damage in a model of progressive MS [55]. This model used non-obese diabetic mice, which have a genetic background of high innate immunity activation and aberrant activation of microglia that leads to an exacerbation of symptoms at the chronic phase [56]. Indeed, microglia depletion in the chronic phase (not tested before) blocked EAE progression [55]. Thus, the results of these studies are not comparable with ours because both the genetic background of the mice and the time window of the treatment are different.

More importantly, microglia depletion exacerbates demyelination and impairs remyelination in a neurotropic coronavirus infection model of MS [57]. The authors showed a higher accumulation of damaged myelin deposits and debris in microglia-depleted mice, which leads to impaired myelin repair and prolonged clinical disease. They propose that microglia functions could not be compensated by infiltrating macrophages. These results, although in a different MS model, are at odds with our data as we did not detect a deficit on myelin clearance, suggesting that in the EAE paradigm used in the current study macrophages compensate and efficiently phagocytose myelin. Accordingly, whereas microglia depletion increases monocyte infiltration in EAE lesions in the spinal cord, which could compensate for the microglia loss, it decreases the number of macrophages in the virus model of MS [57].

To sum up, we provide solid evidence showing that microglia and meningeal macrophages limit during EAE induction the infiltration and dispersion of peripheral macrophages throughout the CNS. Moreover, our results suggest that microglia are not essential for the development of chronic EAE nor for proper remyelination in this model. In addition, we described a microglia-related mechanism promoting early antigen presentation in the CNS, by modulating the expression of B7 co-stimulatory molecules in other APCs, such as DCs or other myeloid populations. This lack of antigen presentation to infiltrating T cells delays the reactivation of these lymphoid cells and therefore provokes a slowdown of the EAE early events.

## Supplementary information

Suplementary Figure 1

Supplemetary figure 1 legend

Reproducibility checklist

## Data Availability

All data generated during this study are included in this published article and its supplementary information files.
