# Supplementary figures and images for: Microglia and meningeal macrophages depletion delays the onset of experimental autoimmune encephalomyelitis
Source: Cell Death Dis. 2023 Jan 12;14(1):16. doi: 10.1038/s41419-023-05551-3 (PMC9835747; doi:10.1038/s41419-023-05551-3)

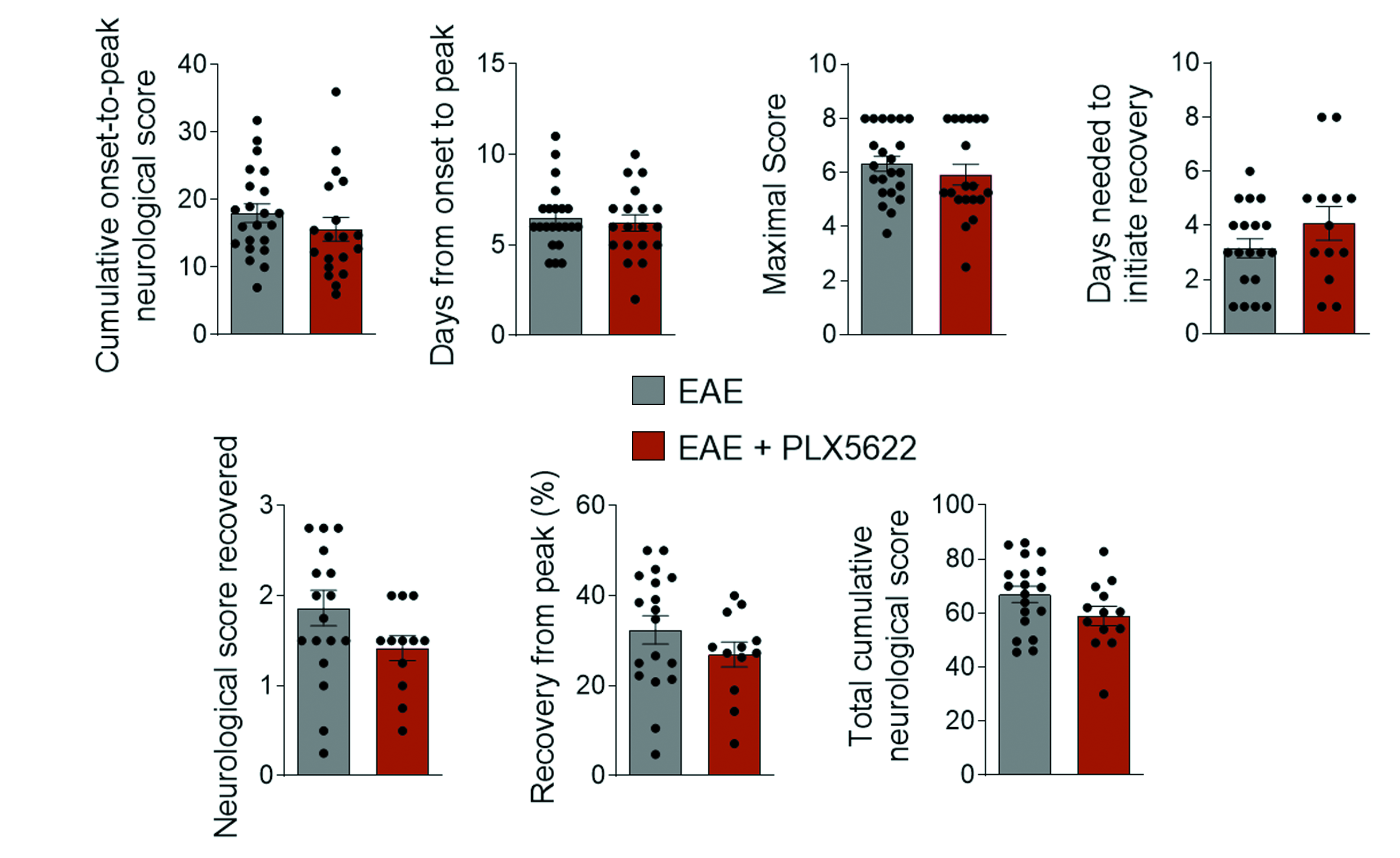

Supplement: Supplementary file 1 — Suplementary Figure 1 [file 41419_2023_5551_MOESM1_ESM.tif]
