# Supplementary material for: Microglia and meningeal macrophages depletion delays the onset of experimental autoimmune encephalomyelitis
Source: Cell Death Dis. 2023 Jan 12;14(1):16. doi: 10.1038/s41419-023-05551-3 (PMC9835747; doi:10.1038/s41419-023-05551-3)
Supplement: Supplementary file 2 — Supplemetary figure 1 legend [file 41419_2023_5551_MOESM2_ESM.docx]

**Supplementary Figure 1.** Clinical parameters related to EAE induction, severity or recovery calculated in control and PLX5622-treated mice at 35 dpi as follows: cumulative onset-to-peak neurological score (sum of all the clinical scores from disease onset to the peak), days from onset to peak (days elapsed from disease onset to peak), maximal score (maximal neurological score shown by each mouse), days needed to initiate recovery  (days elapses from the peak day to the day beginning the recovery), neurological score recovered (difference between maximal neurological score and the score after maximal recovery), recovery from peak (percentage of neurological score recovered from the maximal score) and total cumulative neurological score (sum of all the clinical scores from disease onset to the chronic phase). Data are presented as means ± SEM (n = 20-25 from three independent EAE experiments; neurological score in Fig. 1C).
